# SARS-CoV-2 Pandemic in a Small-Sized Municipality in Ceará State, Brazil: Temporal and Spatial Evolution

**DOI:** 10.3390/tropicalmed9050097

**Published:** 2024-04-26

**Authors:** Jaliana Holanda Nascimento dos Santos, Carlos Henrique Alencar, Jorg Heukelbach

**Affiliations:** 1 Postgraduate Course in Public Health, School of Medicine, Federal University of Ceará, Fortaleza 60.430-140, Brazil; jalianaholanda@gmail.com (J.H.N.d.S.); carllosalencar@ufc.br (C.H.A.); 2 Municipal Health Secretariat of Itapajé, Itapajé 62.600-000, Brazil

**Keywords:** Brazil, control measures, COVID-19, spatial analysis, temporal analysis

## Abstract

Data on the temporal and spatial evolution of SARS-CoV-2 and local control measures and their effects on morbidity and mortality patterns in rural Brazil are scarce. We analyzed the data from case notification systems, epidemiological investigation reports, and municipal decrees in Itapajé, a small municipality in Ceará State in northeast Brazil. For spatial and spatio-temporal analyses, cases and deaths were mapped. There were a total of 3020 cases of COVID-19, recorded between April 2020 and December 2021; 135 (4.5%) died. The cumulative incidence and mortality rates were 5650.3 cases and 252.6 deaths per 100,000 people, respectively. The index case of SARS-CoV-2 in Itapajé was diagnosed in March 2020. The first peak of cases and deaths occurred in May 2020. The second wave peaked in May 2021, with the highest number of deaths in March 2021. According to the spatial analysis, the highest density of cases and deaths occurred in the central urban areas. In these areas, there were also the clusters of highest risk according to the spatio-temporal analyses. The municipal government issued 69 decrees on restriction measures, surveillance, and the maintenance of social isolation as a response to the pandemic. The spread of the SARS-CoV-2 pandemic in Itapajé mirrored the dynamics in large metropolitan regions, going from central neighborhoods of low socio-economic status to the wealthier peripheries.

## 1. Introduction

SARS-CoV-2 was first identified in Brazil in February 2020, and thereafter disseminated throughout the large metropolitan areas of the state capitals. São Paulo, in the southeast, was the main disseminator of SARS-CoV-2 during the first three weeks of the pandemic. Shortly thereafter, 16 other cities contributed intensively to the dissemination of cases across the country [1]. The virus then spread further to the interior of the country. By 22 March 2020, 25 days after the first confirmed case, all states had reported COVID-19 cases [2].

In Ceará State in northeast Brazil, the first cases were confirmed on 15 March 2020 [3]. One month later, the state capital Fortaleza and its metropolitan region had high detection rates. Other regions in the rural hinterland were also registering high detection rates, confirming the spread of the disease in the state [4]. By April 2020, 57% of the 184 municipalities in Ceará had confirmed cases. By May 2023, a total of 1,470,075 cases of COVID-19 and 28,179 deaths were confirmed in the state, with a case fatality rate of 1.9% [5]. In the same period, Brazil recorded a total of more than 37 million cases and 700,000 deaths [6].

During the first pandemic waves, the federal government did not perform structured measures to respond to the pandemic at the national level, leaving the states, Federal District, and municipalities alone with decision-making regarding social distancing and other control measures [7]. The COVID-19 vaccination campaign in Brazil began only in January 2021, initially covering priority groups. Despite this delay and little support from the federal government, a total of 513,470,835 vaccine doses were applied by May 2023 due to the efforts of the state governments and the Unified Health System (SUS) [7,8,9]. Ceará State started vaccinations against COVID-19 in January 2021, and by May 2023, reached a two-dose vaccination coverage rate of 90.1% in the general population [6]. A study in Ceará showed a massive reduction in COVID-19-related deaths in vaccinated >75-year-olds [10].

Since the registration of the first cases of COVID-19, the municipality of Itapajé, in the interior of the state, developed control measures. Despite all their efforts, the virus spread in the municipality and caused a collapse of the health services. Here, we describe the temporal and spatial evolution of the pandemic in this small municipality, starting with the COVID-19 index case, and describe the control measures adopted by the local government and their effects on the morbidity and mortality patterns.

## 2. Materials and Methods

### 2.1. Study Area

**Figure 1:**
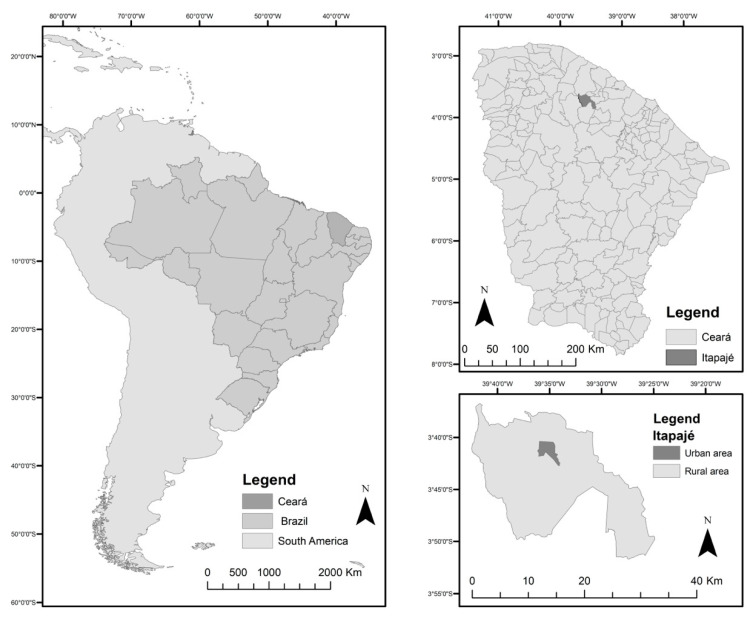
Location of Itapajé, Ceará, Brazil.

The municipality of Itapajé is located in the northern region of Ceará state, 123 km from the state capital Fortaleza (Figure 1). It has an area of 431 km^2^ and is characterized by a semi-arid tropical hot climate, with average yearly rainfall of 858 mm and average annual temperatures ranging between 26 °C and 28 °C.

The estimated population in 2021 was 53,448 inhabitants, with an urbanization rate of 70.3% and a population density of 110 inhabitants/km^2^.

### 2.2. Study Population and Period

The study population was composed of the 3020 confirmed COVID-19 cases, registered between 2 April 2020, the date of confirmation of the first case of COVID-19 in the municipality, and 31 December 2021. We included notifications of confirmed cases residing in the municipality, and excluded duplicate entries and notifications of non-residents.

### 2.3. Variables and Data Sources

Data on the onset of symptoms, addresses, case evolutions, and dates of death were obtained from the local database of the Itapajé Municipal Health Department of the e-SUS Notifica and the Influenza Epidemiological Surveillance Information System (SIVEP-Gripe). E-SUS Notifica is an online system created by the Federal SUS Department of Informatics (DATASUS) to register notifications of suspected and confirmed mild cases of COVID-19. Notifications of severe cases of acute respiratory syndrome (SARS) hospitalized in the public and private networks, and the notification of deaths from COVID-19 regardless of hospitalization were registered in SIVEP-Gripe [11].

Other sources of information included the investigation report of the first case of SARS-CoV-2 detected in Itapajé, the municipal plan for coping with the COVID-19 pandemic, and reports of sanitary surveillance inspections. Additional information was obtained from municipal decrees and the municipal plan for a narrative description of the control measures implemented in the municipality.

Demographic data of the municipality were obtained from the Brazilian Institute of Geography and Statistics (IBGE).

### 2.4. Data Processing and Analysis

The characterization of the first confirmed case of COVID-19 was carried out in a narrative way based on the municipal epidemiological surveillance report.

In the epidemiological curve, the confirmed cases of COVID-19 are presented by the epidemiological week (EW) according to the date of onset of symptoms. The epidemic curve of deaths is presented by date of the occurrence of death.

Based on the absolute number of confirmed cases and deaths by COVID-19 and population data, we calculated the incidence rates (the number of confirmed cases divided by the resident population, multiplied by 100,000 inhabitants), mortality rates (the number of deaths by COVID-19 divided by the resident population, multiplied by 100,000 inhabitants), and case fatality rates (the number of deaths by COVID-19 divided by the total of confirmed cases, multiplied by 100).

We created geographic location maps (latitude and longitude) of the cases and deaths for each of the pandemic waves. The maps of cases and deaths were obtained by locating the address of each case and death in the urban area of the city using Google Earth software. Each address was geographically located in .kml format and then converted into .shp format, and adjusted to Polychonic/SIRGAS 2000 projection.

In addition, we used a map of census sectors of the urban area of the municipality of Itapajé with the information of the resident population in each of these sectors. The map of the census sectors was obtained from the IBGE site for the year 2021 (https://www.ibge.gov.br/geociencias/organizacao-do-territorio/estrutura-territrial/26565-malhas-de-setores-censitarios-divisoes-intramunicipais.html?=&t=acesso-ao-produto (accessed on 21 March 2021)) in .kml format and converted into .shp, and its projection was adjusted. The population data were obtained from the local IBGE office and were merged with the sector map, creating a geographic database.

Kernel density maps were created using Terraview 4.2.2 software (INPE—National Institute for Space Research; http://www.dpi.inpe.br/terralib5/wiki/doku.php?id=wiki:downloads:terraview_terralib_4.2.2 (accessed on 21 March 2021)). The following parameters were used: For the support region, the grid option of 1000 columns was chosen. The base was the map of the census sectors of the urban area of the city, and the data sets were the maps of the cases and deaths during each of the pandemic waves. In both analyses, the algorithm used was the quadratic function to calculate the density and adaptive radius. The quadratic function gives more weight to the closest values, almost completely disregarding the value of more distant points, showing a gradual decrease as the distance changes. This function also defines the density categories.

For the calculation of the kernel ratio, the support region was gridless. The first data set was composed of the maps of the cases and deaths during each of the waves, and the second data set was the map of the census sectors; an attribute of the latter was the population of each sector. Similarly to the kernel density analysis, the algorithm used was with the quadratic function to calculate the density and adaptive radius.

For the spatio-temporal analyses, the incidence and mortality coefficients were calculated for each census tract. SaTScan software version 10.1 was used to process and analyze the data. The units of analysis were the municipality’s 47 urban census tracts and their respective geographical coordinates. A retrospective spatio-temporal analysis was used to identify the high-risk areas for COVID-19 incidence and mortality. The following parameters were considered: the Poisson probabilistic model, statistical inference with 99,999 Monte Carlo replications, 50% of the population at risk, a maximum cluster size of a 0.5 km radius, and *p*-value < 0.05. Clusters with only one sector were excluded. The output file in “.gis” format was used to georeference the information and create thematic maps. The digital map of the urban area of Itapajé in shapefile format (.shp) was used as the basis for creating the maps, with the colors standardized by gradual style, according to the relative risk value of each cluster. QGIS software version 3.28.0 Firenze was used to build the thematic maps.

### 2.5. Ethical Aspects

The data were extracted from secondary databases. The use of the data was authorized by the Municipal Health Secretariat of Itapajé. This study was approved by the Research Ethics Committee (CEP) of the Universidade Federal do Ceará (UFC) under CAAE 51850221.8.0000.5054.

## 3. Results

### 3.1. Index Case

The first diagnosed COVID-19 case in Itapajé was a 61-year-old housewife. She had traveled to São Paulo to visit relatives on 1 March 2020, where she stayed until 17 March 2020. On 18 March 2020, she returned to Itapajé. She sought medical assistance at the local hospital on 20 March 2020 with complaints of a sore throat, dry cough, running nose, fever, and dyspnea. She reported the onset of signs/symptoms on 14 March 2020. The patient was immediately treated and reported as a suspected case of COVID-19, and was referred to the state reference hospital for infectious diseases in Fortaleza, the state capital. There, she received medical care and a naso-oropharyngeal swab was collected for real-time RT-PCR examination, with confirmation of SARS-CoV-2 on 2 April 2020. She progressed to complete cure.

### 3.2. Pandemic

Since the confirmation of the first allochthonous case in Itapajé, 3020 cases were registered by 31 December 2021. Of these, 135 (4.5%) progressed to death. The cumulative incidence and mortality rates were 5650.3 cases and 252.6 deaths per 100,000 inhabitants, respectively.

**Figure 2:**
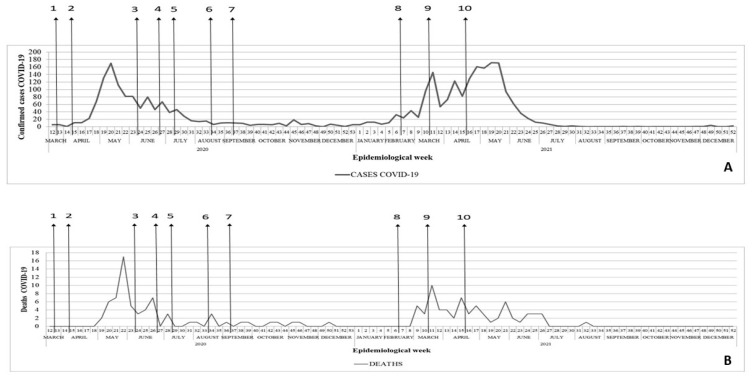
(**A**) Epidemiological curve of confirmed cases of COVID-19 by onset of symptoms and by epidemiological week. (**B**) Epidemiological curve of deaths by epidemiological week. Vertical arrows indicate municipal control measures: (1) First restrictive measures (17 March 2020); (2) intensification of restrictive measures (1 April 2020); (3) loosening of restrictive measures—opening of industries and civil construction chains (31 May 2020); (4) reopening of non-essential businesses and services (22 June 2020); (5) reopening of restaurants and similar (6 July 2020); (6) release of gyms and religious ceremonies (10 August 2020); (7) return of face-to-face educational activities (31 August 2020); (8) restrictive measures (18 February 2021); (9) strict social isolation (13 March 2021); (10) relaxation of strict social isolation (11 April 2021).

The first autochthonous cases of COVID-19 in Itapajé occurred in mid-March 2020, with a significant increase in late April and early May, and the peak of cases during this first wave of transmission in May 2020 (170 cases) (Figure 2). This was followed by a reduction in June and a stabilization of the number of cases at low levels until December 2020 (Figure 2). The second pandemic wave peaked in May 2021 (172 cases). This was followed by a reduced number of cases until June, when transmission dropped to very low levels.

The first deaths from COVID-19 occurred in May 2020, 80 days after the onset of the first cases. The peak number of deaths occurred in May, followed by a reduction during the months thereafter. During the second wave, deaths were recorded from February to early July, with the peak in March (Figure 2).

The first pandemic wave resulted in incidence and mortality rates of 2302.7 cases and 126.3 deaths per 100,000 inhabitants, respectively. The case fatality rate during this period was 5.5%. During the second wave, which began by the end of December 2020, the incidence was 3364.0 cases per 100,000 inhabitants, and the mortality was 127.2 deaths per 100,000 inhabitants, with a case fatality rate of 3.8%.

### 3.3. Spatial Distribution

**Figure 3:**
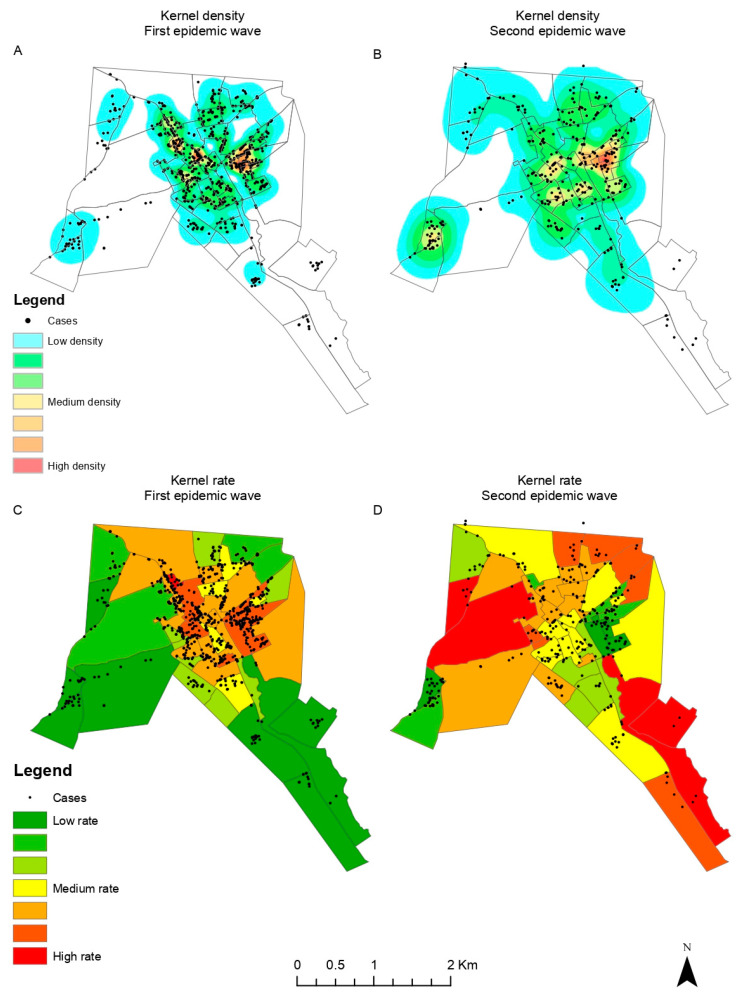
COVID-19 cases: (**A**) kernel density, first pandemic wave; (**B**) kernel density, second pandemic wave; (**C**) kernel ratio, first pandemic wave; (**D**) kernel ratio, second pandemic wave.

**Figure 4:**
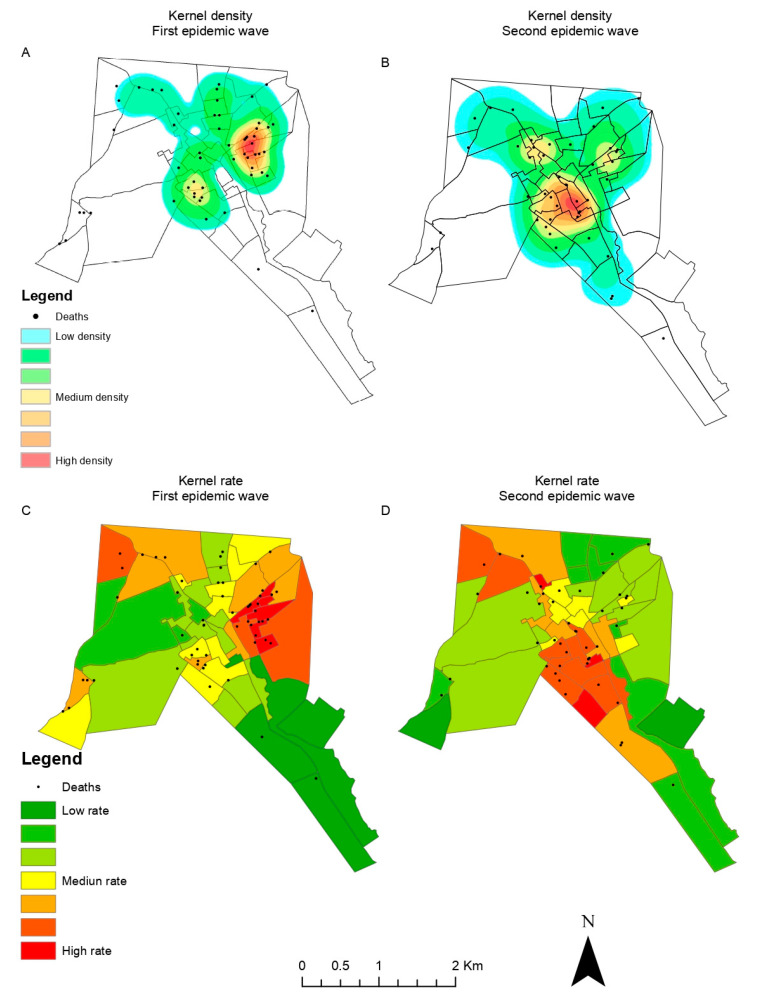
COVID-19 deaths: (**A**) kernel density, first pandemic wave; (**B**) kernel density, second pandemic wave; (**C**) kernel ration, first pandemic wave; (**D**) kernel ratio, second pandemic wave.

A total of 2691 confirmed COVID-19 cases and 132 deaths were geographically located and mapped. The kernel density analysis revealed higher intensities of cases and deaths by COVID-19 located in the city center of Itapajé (Figure 3 and Figure 4).

In the beginning of the pandemic (first wave), cases were concentrated in the central area of the city, with a subsequent expansion to the peripheral wealthier neighborhoods during the second wave (Figure 3). A similar pattern could be observed for high-risk death clusters (Figure 4).

### 3.4. Spatio-Temporal Analyses

**Figure 5:**
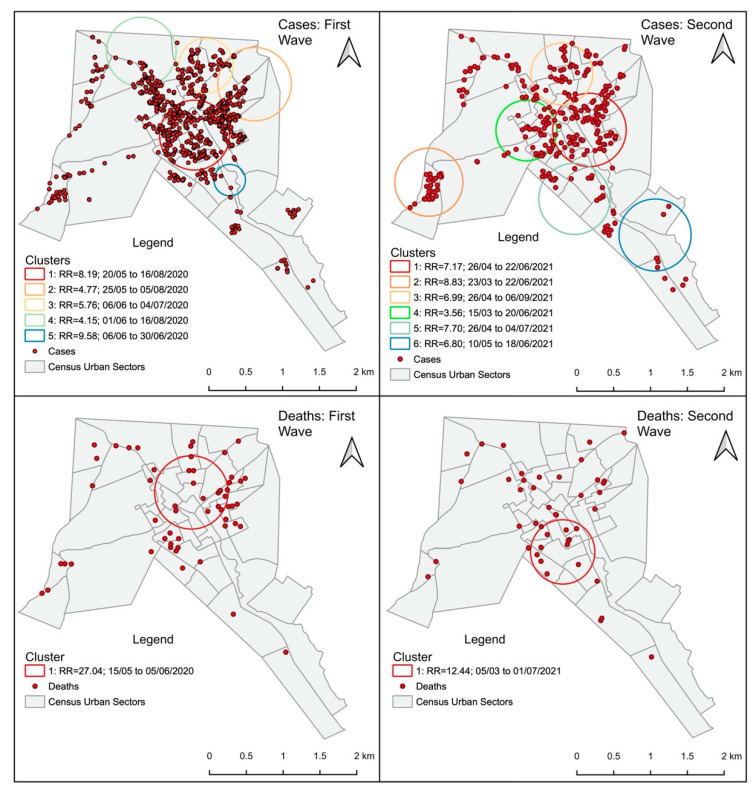
Spatio-temporal analyses of COVID-19 cases and deaths in the first and second pandemic waves.

In the first pandemic wave, we identified five case clusters and one death cluster. The case clusters occurred mainly between May and August. Cluster 1, with 14 sectors, showed the longest period of activity for almost 14 weeks and a relative risk (RR) of 8.19 (*p* < 0.001). Cluster 5 showed an RR of 9.58 (*p* < 0.001); with only two sectors, it included the second largest population at risk and remained active for 25 days. The death cluster had an RR of 27.04 (*p* < 0.001) and remained active between 15 May and 5 June 2020. The central region of the city was the main area affected, with the most significant case and death clusters (Figure 5).

The second pandemic wave showed a similar pattern to the first wave, with the central area of the city mostly affected by COVID-19. Case Cluster 2, located in the west of the city, had an RR of 8.83 (*p* < 0.001), which was active for three months and had a population of 1670 inhabitants at risk. The cluster of deaths shifted slightly to the south compared to the first wave and had an RR = 12.44, remaining active for almost four months (Figure 5).

### 3.5. Control Measures

The municipality published a total of 69 decrees that included restriction measures, surveillance, and the maintenance of social isolation to control the spread of the pandemic. The first decree, published on 17 March 2020, announced a state of health emergency in the municipality and established restrictive measures, such as the suspension of any public or private events, activities in public schools, gyms, concert halls, municipal stadiums, and commercial establishments with more than 30 people, and collective activities and events in temples and churches. Then, through another decree from 1 April 2020, the municipality intensified the restriction measures by closing down non-essential businesses and industries. On 6 May 2020, the obligatory use of industrial or household face masks was established throughout the municipal territory. Starting 31 May 2020, the restrictive measures were gradually loosened.

With the start of the second pandemic wave, on 15 February 2021, the state of public emergency was extended in the municipality. On 18 February 2021, additional measures were announced: suspension of classes and classroom activities in educational establishments; restriction on opening hours of businesses and services; prohibited use of public spaces from 5:00 p.m. to 5:00 a.m.; adoption of a remote work regime for the municipal civil service with the exception of essential services; and institution a “curfew”. On 13 March 2021, strict social isolation was instituted with the establishment of a special duty of confinement, control of vehicle circulation, prohibition of the circulation of people in public spaces and on public roads, prohibition of face-to-face religious celebrations, and the closure of non-essential businesses. The rigid lockdown began to be gradually relaxed on 11 April 2021 (Figure 2).

Vaccination against COVID-19 began in the municipality on 20 January 2021, prioritizing public health professionals who worked in the front line, and ≥75 year-olds. In March, vaccinations were extended for those aged 65 to 74 years; in April, for 60- to 64-year-olds; and in June, for the population with comorbidities in descending order of age and public and private school teachers. Thereafter, the vaccination campaign included the population aged 5 to 59 years (general population), according to the decreasing order of age groups. In the year 2021, the vaccination coverage reached 40.6%.

## 4. Discussion

Our study described the pandemic characteristics, control measures, and spatio-temporal distribution of COVID-19 in a small municipality in rural northeastern Brazil. The city borders a federal highway with a high flow of people and goods, connecting the state capital to cities in the interior of the state and other states, and it is a commercial center for small neighboring towns. This mobility possibly contributed to the introduction and maintenance of SARS-CoV-2. Previous studies have reported the highest incidences in urban metropolitan regions, related to their higher populations, higher population density, and high urban mobility [12,13,14,15].

The epidemiological curve of COVID-19 shows a pattern similar to that observed in the state of Ceará and in the capital Fortaleza [16,17]. In both pandemic waves, the peaks of cases and deaths in the capital preceded, by a few weeks, those observed in Itapajé and in the interior of the state. In fact, evidence suggests that the northeastern Brazilian states share the same epidemiological characteristics, extending from capital cities to the rural hinterland municipalities [18]. Our findings also confirm previous studies on the state capital Fortaleza—a major metropolitan city—where the pandemic started in wealthier neighborhoods and then spread to the more socially vulnerable neighborhoods [19,20].

Itapajé had a lower incidence of COVID-19 per 100,000 inhabitants than those obtained in Brazil (10,636.7), Ceará State (10,527.0), and the state capital Fortaleza (10,376.2) [5]. However, in contrast to major cities, the health system did not have adequate physical infrastructure or human resources to deal with the ongoing pandemic. There was only a single secondary-level hospital with a capacity of 30 beds. Consequently, the mortality rate was higher, as compared to Brazil (292.4) and Ceará State (270.0), presenting similar values in the first and second waves of infection [6]. Similarly, the case fatality in Itapajé was higher than in Ceará (2.6), and the state capital Fortaleza (4.0) [5]. The scarcity of health service support, especially the limited availability of intensive care unit (ICU) beds, associated with the rapid spread of SARS-CoV-2, may have contributed to the higher mortality and case fatality rates of COVID-19 in smaller municipalities [21].

The spatial analyses showed that the highest density and the most important clusters of cases occurred in the central area of the city, where there is a greater flow of workers and people in search of commerce and services, which made viral transmission possible [22]. Mobility restrictions may have slowed the spread of SARS-CoV-2, but they could not change the routes of expansion of the virus [23].

Similar to many other infectious diseases, the occurrence of COVID-19 is related to socio-economic variables [24]. In line with this, in our study, spatio-temporal analyses confirmed that the highest risks occurred during clear-cut periods in municipal areas of lower socio-economic strata. Most vulnerable neighborhoods have deficient urban infrastructure, high unemployment rates, difficult access to basic health services, and lack of household structure, making it difficult to adopt measures to control the disease [25]. Similarly, higher risks of deaths from COVID-19 have been reported in resource-poor areas and in regions with high population density and urban mobility and deficient health services [26].

Social vulnerability, informal work, unemployment, and extreme poverty were factors that probably also interfered with adherence to the control measures [18]. People with informal work, such as market vendors, were forced to go out daily in search of income, exposing themselves to infection. Thus, the challenge in implementing social distancing measures was to balance the desired effects on health with the inevitable economic and social damages [27]. This required ensuring policies of social protection, minimum income, and labor protection. Consequently, state and municipal governments exempted the most vulnerable population groups from electricity and water bills, and they distributed food baskets [28]. On the other hand, government aid related to the health crisis may have contributed to a decrease in formal employment [29]. At the national level, the National Congress approved emergency aid to ensure a minimum income to the vulnerable population and to guarantee the social distancing measures. However, a large part of the population needed to crowd in long lines at the local bank or lottery to receive these benefits. This situation was also observed in Itapajé and, considering that the city is a hub for banking services for small neighboring communities, the consequent increase in local mobility may have also impacted the spread of the pandemic. Additional interventions should include not only more intensive surveillance but also measures aimed at reducing risks caused by socio-economic inequalities, for example, improved housing, access to health services, and health systems strengthening [24,25,29]

The lack of leadership from the federal government in formulating a national response to mitigate and suppress COVID-19 led the Federal Supreme Court (STF) to assign to the states, Federal District, and municipalities the competence to coordinate responses in their territories. Thus, the Government of Ceará took over the coordination of these measures in the state, guiding the municipalities in prevention and control [30]. Thus, the first social distancing measures to combat the spread of SARS-CoV-2 were implemented in Itapajé before the notification of the first case in the municipality.

Another relevant factor for the spread of the disease was the denial of the serious health situation by some population groups and the consequent noncompliance with the measures of social distancing. The denialist posture of the federal government clearly did not contribute positively to the efforts of the state and municipal managers focusing on mitigation of the pandemic [31].

The relaxation of social distancing measures in Itapajé during the first wave occurred when the epidemiological curve showed an inconsistent decrease in the number of cases and a considerable number of deaths. In fact, the moment of relaxation of social distancing measures in the northeastern capitals occurred prematurely. Despite these limitations of the measures adopted by states and municipalities in the northeast, there is evidence that the negative effects of the pandemic were mitigated [15,32].

The reopening of shops and the municipal elections that took place in November 2020 influenced the onset of the explosive growth of cases during the second wave of COVID-19 in northeastern Brazil [18]. In Itapajé, the municipal elections and the change in management caused discontinuity in the epidemiological monitoring and the actions to contain the pandemic due to the relaxation of control measures in the pre-electoral period.

In Brazil, there was a delay in the acquisition of vaccines against COVID-19, and the campaign started only in January 2021. The number of doses were initially insufficient to cover the entire population, requiring the prioritization of high-risk population groups and subsequent expansion to the general population, as more and more doses of vaccine were acquired. The vaccination campaign in Itapajé progressed slowly, and there was a low vaccination adherence in a considerable part of the population. The local health system outlined several strategies, such as task forces, drive-through facilities, active search in homes, industries, and public agencies, finally achieving a vaccination coverage of 72.2% by May 2023 for the first dose, and of 70% for a second/single dose [5]. Despite all these difficulties, the Unified Health System (SUS) managed to increase accessibility to the vaccine in the entire Brazilian territory, achieving success of the vaccination campaign, and contributing to reducing the occurrence and transmission of the disease and the number of deaths [7,10]. SUS played a pivotal role in the planning, coordination, and execution of mitigation measures at the municipal level. SUS guarantees the promotion, protection, and recovery of health to the Brazilian population, guided by the principles of universalization, equity, and integrality.

The limitations of our study include the probable underreporting of cases and deaths at the beginning of the pandemic due to the scarcity of tests and reagents that led to the initial recommendation to test only patients >60 years, people with comorbidities, and those with severe conditions admitted to hospital units. During the second wave, the better availability of diagnostic tests increased the quality of the official data from the epidemiological surveillance, enabling an approximation of the size of the pandemic at the local level. This may be related to the higher incidence rate of COVID-19 per 100,000 inhabitants recorded in this wave of infection in Itapajé. As individual data on socioeconomic variables, access to health services, and vaccination coverage for COVID-19 were not available, the effects of these variables could not be analyzed at the individual level and should be the object of further study.

## 5. Conclusions

The spread of the SARS-CoV-2 pandemic in Itapajé mirrored the behavior observed in large metropolitan regions, from central neighborhoods of low socio-economic status to the wealthier peripheries. The spread was influenced by social vulnerability, insufficient health infrastructure, urban mobility, control measures adopted, and the deficiency of the health services. It is necessary to strengthen the health system and epidemiological and sanitary surveillance, expand the access and coverage of medium- and high-complexity services in small municipalities, and strengthen vaccination programs. Our findings are relevant for the understanding of future pandemics in small municipalities since most studies conducted at the national level cover states and major metropolitan areas.

## Data Availability

The data presented in this study are available upon request from the corresponding author. The data are not publicly available due to privacy.
